# The complete mitochondrial genome of *Megaderma lyra* (Indian false vampire)

**DOI:** 10.1080/23802359.2018.1443851

**Published:** 2018-02-26

**Authors:** Mengjia Liu, Yanhong Lan, Yi Cao

**Affiliations:** Microbiology and Metabolic Engineering of Key Laboratory of Sichuan Province, College of Life Science, Sichuan University, Chengdu, China

**Keywords:** *Megaderma lyra*, mitochondrial genome; protein-coding genes

## Abstract

The Indian false vampire (*Megaderma lyra*), known as the greater false vampire bat, the Indian false vampire bat, and the greater false-vampire, is typical echolocation mammals. It has been listed in the IUCN Red List of threatened species and included in the Red Book of Endangered Animals in China. Herein, we described 17,055 bp of *M. lyra* mtDNA that includes 13 protein-coding genes (PGCs), two rRNA genes (12S rRNA and 16S rRNA), 22 transfer RNA (tRNA) genes, and one control region (D-loop). The complete mitochondrial genome sequence will provide new molecular biology information to further understand the genetic diversity of the *M. lyra* and to protect this population.

The Indian false vampire (*Megaderma lyra*) belongs to the family *Megadermatidae* that is widespread throughout South Asia and Southeast Asia (Csorba et al. 2008). It is a typical echolocation mammal, and can hunt using both vision and passively listening for its prey, and has also been observed catching prey in complete darkness without echolocation (Reilly 2010, Mohd-Azlan et al. 2016). However, molecular studies about *M. lyra* are limited, what’s more, no mitochondrial genome of *M. lyra* is available until now. Here, we assembled and characterized the complete mitochondrial genome of *M. lyra*.

The total genomic DNA of *M. lyra* was isolated from US036, and extracted from the blood of an adult *M. lyra*, then sequenced with the Genome-wide short-read sequencing (Illumina HisSeq 200, 500 bp insert size, 2 × 90 bp pair-end) (Zhang et al. 2014), the information of sample of *M. lyra* was stored in NCBI (Accession No. SAMN02212695) and the SRA Accession Number is SRS454316. The sequencing reads were trimmed using Trimmomatic v0.36 (Bolger et al. 2014), and assembled with NOVOPlasty v2.6.3 software (Dierckxsens et al. 2017), then annotated and generated a physical map by MitoFish 3.30 (http://mitofish.aori.u-tokyo.ac.jp/) (Iwasaki et al. 2013).

The complete mitochondrial genome of *M. lyra* is a double-stranded, circular DNA 17,055 bp in total length (GenBank Accession No. MG586969), and includes 13 protein-coding genes, 2 ribosomal RNA genes (12S rRNA and 16S rRNA), 22 tRNA genes, and one control region (D-loop). The overall nucleotide composition was 30.15% A, 28.32% T, 15.37% C, and 26.13% G, respectively, and the percentage of G + C content was 41.51%. Twelve of the PCGs use complete (TAA) or incomplete (T––) stop codon. The 12S rRNA and 16S rRNA genes are 968 and 1564 bp, respectively. The lengths of 22 tRNA genes range from 59 bp (*tRNA-Ser*) to 75 bp (*tRNA-Leu*). The D-loop length is 1151 bp and lies between the *ATPase 8* and *tRNA-Phe* genes.

The Phylogenetic analysis of 13 mitochondrial genomes using MEGA 7 (Kumar et al. 2016) shows that *M. lyra* and *M. psilurus* are the most closely related species (Figure 1). The mitogenome of *M. lyra* would contribute to the understanding of the phylogeny and evolution of Rodentia.

**Figure 1.**
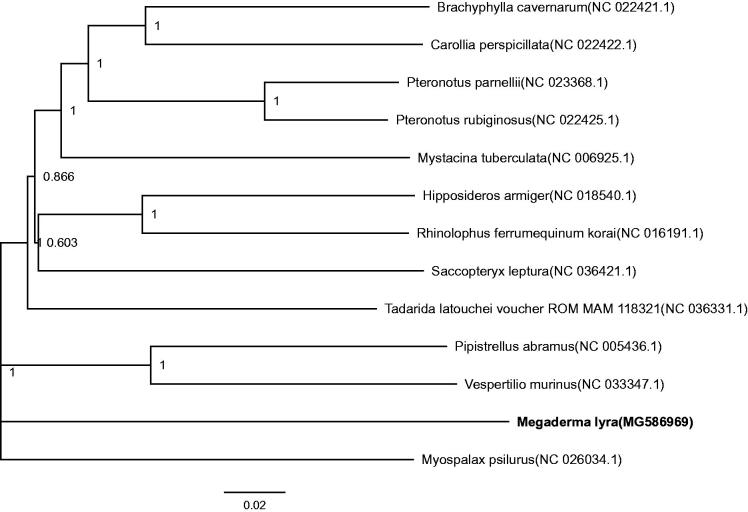
Phylogenetic tree constructed with *M. lyra* and 12 other species mitogenomes and this species belong to Microchiroptera. It was constructed based on the alignment of maximum-likelihood method within the MEGA 7. The bootstrap support values are generated using 1000 replications. GenBank sequences are listed, followed by species names.
